# Exome sequencing identifies somatic mutations in novel driver genes in non-small cell lung cancer

**DOI:** 10.18632/aging.103500

**Published:** 2020-07-06

**Authors:** Manman Zhang, Lele Zhang, Yan Li, Feng Sun, Ya Fang, Ruijia Zhang, Jin Wu, Guanbiao Zhou, Huaidong Song, Liqiong Xue, Bing Han, Cuixia Zheng

**Affiliations:** 1 Clinical Research Center, Shanghai Ninth People's Hospital, Shanghai Jiao Tong University School of Medicine, Shanghai, China; 2 Department of Endocrinology, Shanghai Ninth People’s Hospital, Shanghai Jiao Tong University School of Medicine, Shanghai, China; 3 Department of Pulmonary Medicine, Shanghai Chest Hospital, Shanghai Jiao Tong University School of Medicine, Shanghai, China; 4 State Key Laboratory of Molecular Oncology, National Cancer Center, National Clinical Research Center for Cancer, Cancer Hospital, Chinese Academy of Medical Sciences and Peking Union Medical College, Beijing, China; 5 Department of Oncology, Dongfang Hospital, Tongji University School of Medicine, Shanghai, China; 6 Department of Respiration, Yangpu Hospital, Tongji University School of Medicine, Shanghai, China

**Keywords:** non-small-cell lung cancer, UNC5D, exome sequencing, driver gene mutation

## Abstract

Lung cancer is the leading cause of cancer death worldwide and accounts for more than one-third of all newly diagnosed cancer cases in China. Therefore, it is of great clinical significance to explore new driver gene mutations in non-small-cell lung cancer (NSCLC). Using an initial bioinformatic analysis, we identified somatic gene mutations in 13 patients with NSCLC and confirmed these mutations by targeted sequencing in an extended validation group of 88 patients. Recurrent mutations were detected in *UNC5D* (7.9%), *PREX1* (5.0%), *HECW1* (4.0%), *DACH1* (2.0%), and *GPC5* (2.0%). A functional study was also performed in UNC5D mutants. Mutations in *UNC5D* promoted tumorigenesis by abolishing the tumor suppressor function of the encoded protein. Additionally, in ten patients with lung squamous cell carcinoma, we identified mutations in *KEAP1/NFE2L2* that influenced the expression of target genes *in vivo* and *in vitro*. Overall, the results of our study expanded the known spectrum of driver mutations involved in the pathogenesis of NSCLC.

## INTRODUCTION

Lung cancer is the leading cause of cancer death worldwide and accounts for more than one-third of all newly diagnosed cancer cases in China [1]. In general, lung cancer can be divided into two groups, namely, small cell lung cancer and non-small-cell lung cancer (NSCLC). NSCLC, which includes lung adenocarcinoma (ADC) and lung squamous cell carcinoma (SCC), represents approximately 85% of all cases [2] and can be further divided into subgroups according to specific gene mutations [3]. These genes can guide treatment decisions, as targeted therapies have been successfully used in clinical work with impressive results [4–7]. Mutations in 10 driver genes have been identified in patients with lung ADC that contribute to its pathogenesis [8].

Next-generation sequencing (NGS) enables large-scale analyses of DNA sequence alterations in human tissue and was used to identify additional genes related to the pathogenesis of lung cancer and potential therapeutic targets. Recently, an exome-sequencing study identified recurrent mutations in *CREBBP*, *EP300*, and *MLL* that encode histone modifiers and showed evidence for the inactivation of TP53 and RB1 in small-cell lung cancer [9]. The traditional view was that although various subtypes of NSCLC have unique and shared clinical and histological features, such as smoking being the main risk factor for NSCLC, approximately 10 – 15% of all adenocarcinomas arise in never smokers, but their main genetic mutations are similar. [10] Campbell et al examined exome sequences and copy number profiles of 660 lung ADC and 484 lung SqCC tumor/normal pairs and found that recurrent alterations in lung SqCCs were more similar to other squamous carcinomas than to lung ADCs [11]. This finding suggests that the pathogenic genes between lung squamous cell carcinoma and lung adenocarcinoma of different pathological types are different, and lung cancer of the same histopathological type may carry different pathogenic driving genes leading to the carcinogenesis of certain types of cells, which requires the use of different targeted drugs for treatment. Therefore, the search for new pathogenic mutations and driving genes of NSCLC and the further exploration of its pathogenesis may provide important guidance for clinical diagnosis and targeted therapy to promote personalized precision medicine of cancer treatment.

In the present study, we performed whole-exome sequencing of paired frozen tumor and adjacent noncancerous tissues from six SCC patients. Furthermore, we also included whole-genome sequencing data of four SCC patients and three ADC patients from the Zhou lab [12]. The workflow is shown in Supplementary Figure 1.

## RESULTS

### Next-generation sequencing in NSCLC

To control for tumor heterogeneity and passenger mutations, the tumors included in our study all harbored *TP53* mutations, which are known to play an important role in the tumorigenesis of lung epithelial cells. The average sequencing coverage of whole-exome sequencing was reported previously [13].

**Figure 1:**
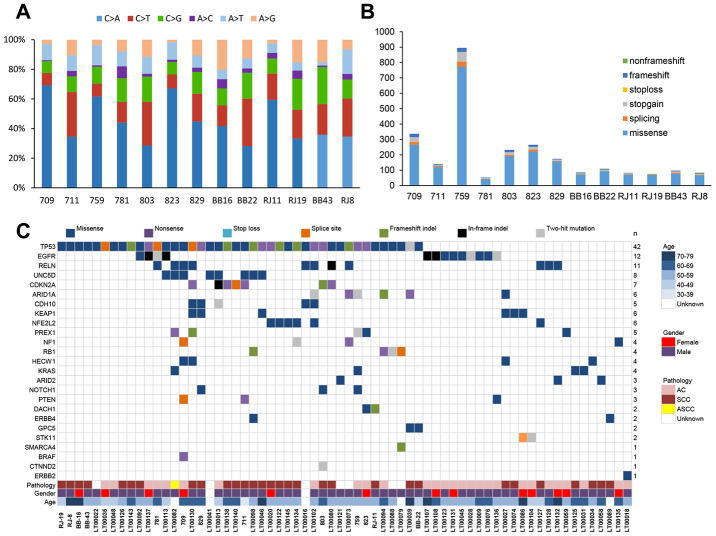
**Next-generation sequencing in NSCLC.** (**A**) Percentages of non-silent somatic single nucleotide variants identified by next-generation sequencing (whole-genome/whole-exome/targeted sequencing) in 13 NSCLC patients. (**B**) Numbers and types of non-silent somatic mutations. (**C**) Mutations identified by next-generation sequencing in 13 NSCLC patients and in a validation group of 88 NSCLC patients. The type of each mutation is shown for every sample, and the number of subjects (n) with mutations is listed on the right. AC, lung adenocarcinoma. SCC, lung squamous cell carcinoma. ASCC, lung adenosquamous carcinoma.

Through a series of bioinformatic analyses (including somatic mutation selection, filtration of nonpathogenic single nucleotide polymorphisms from dbSNP 135, and single nucleotide variants from the ESP 6500 database), we observed that C>A/G>T alterations were more frequent than other forms (Figure 1A), which is similar to the somatic single nucleotide variant spectrum of NSCLC reported in other studies [14]. The somatic non-silent mutation load per subject varied remarkably (mean, 170; range, 61–953). In total, we identified 2,703 non-silent somatic mutations, including 2,285 missense, 191 nonsense, 103 splice site, 108 frameshift, and 16 non-frameshift indels in 2,217 genes (Figure 1B) (Supplementary Table 1). Among these genes, mutations in *TP53*, *CDKN2A*, *PTEN*, *KEAP1*, *NF1,* *RELN*, *KRAS*, and *CDH10* have been reported in NSCLC in previous studies [14–16].

### Mutation validation

Because of the small sample size, we could not perform a MutSigCV [17] bioinformatics analysis to identify potential driver genes. Therefore, we developed an original bioinformatic algorithm to identify driver genes in NSCLC [13]. We first compared the mutation rates of the 2,217 genes identified in the 13 NSCLC patients with those in whole-exome sequencing data from 87 controls (made in-house) and NSCLC patients from the Catalog of Somatic Mutation in Cancer (COSMIC). We found 152 genes that had significantly higher mutation rates (*P* < 0.05, Fisher’s test) in NSCLC patients than in the normal controls (Supplementary Table 2, Supplementary Figure 1). Among these genes, six (*UNC5D, PREX1, HECW1, DACH1, GPC5*, and *CTNND2*) related to oncogenesis according to previous literature [18–22] were selected as candidate driver genes for targeted sequencing in a validation group of samples from 88 NSCLC patients along with 21 genes with mutation rates that were higher than in the COSMIC database and are known driver genes of lung cancer (seven of which were included in the list of 152 genes) (Supplementary Figure 1).

A pool of multiplex PCR primers was designed for target amplification. Paired-end sequencing by an Illumina HiSeq 2500 system was applied to the 88 paired samples with an average coverage of 1,000× (Supplementary Table 3). We identified a total of 462 variants in the 27 candidate genes (Supplementary Table 4). Of these variants, we selected variants that (i) were identified by targeted sequencing in replication experiments or (ii) were called only in one of the experiments, where the sequencing coverage was <25× in replication experiments. One hundred ninety-two of these variants were subjected to Sanger sequencing, which verified 116 somatic mutations (Supplementary Table 5) for a concordance rate with next-generation sequencing of 60.4% (116/192). With this method, four of the six candidate driver genes were confirmed, with *UNC5D* being the most frequently mutated (7.9% (8/101)) followed by *PREX1* (5.0% (5/101)), *HECW1* (4.0% (4/101)), and *GPC5* (2.0% (2/101)) (Figure 1c). Among the known driver genes, *TP53* (41.6% (42/101)), *EGFR* (11.9% (12/101)), *RELN* (10.9% (11/101)), *CDKN2A* (6.9% (7/101)), *ARID1A* (5.9% (6/101)), *KEAP1* (5.9% (6/101)), *NFE2L2* (5.9% (6/101)), and *CDH10* (5.0% (5/101)) were commonly mutated genes. In addition, we also compared the mutation frequency across different large-scale genomic study cohorts with lung adenocarcinoma or squamous cell lung cancers [15–16, 23–25]. The results indicated that the Chinese cohorts were significantly different from the other cohorts with regard to the presence of somatic driver mutations (Supplementary Figure 2).

### Mutations detected in UNC5D

**Figure 2:**
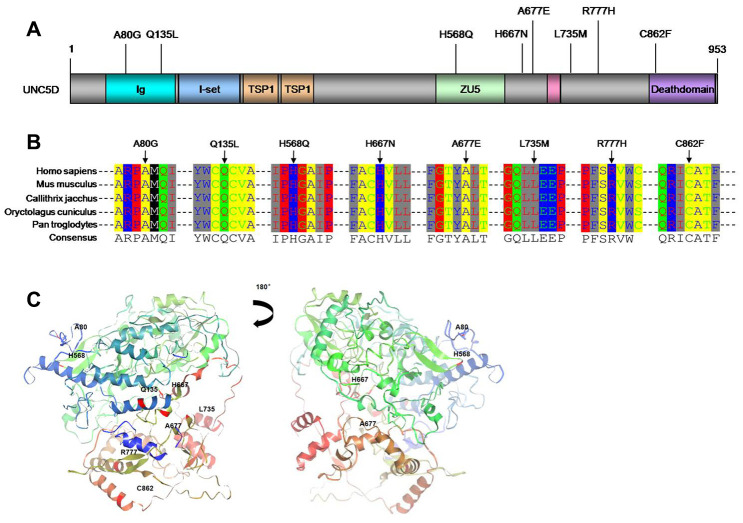
***UNC5D* mutations in NSCLC.** (**A**) Schematic locations of the *UNC5D* mutations. All the mutations were missense mutations. (**B**) Sequence alignment of *UNC5D* across different species. (**C**) Spatial structure prediction of UNC5D. The crystal structure of UNC5D was constructed by I-TASSER from the Zhang Lab. Mutations sites are also labeled.

The *UNC5D* variants comprised eight novel somatic mutations. We utilized SIFT/PolyPhen to predict the functional alterations of these variants, as well as the others identified among the 27 candidate genes (Supplementary Table 5). The eight *UNC5D* variants were all missense mutations (Figures 1C and 2A). Moreover, Ortholog analysis indicated that these missense mutations were located at well-conserved amino acid positions across distinct species, suggesting that these mutations might influence protein function. We constructed the crystal structure of UNC5D by using I-TASSER from the Zhang lab (Figure 2C) [26]. The C862F mutation is located in the death domain of UNC5D, for which alterations have been shown to influence protein function and induce tumorigenesis [20].

### Functional analysis of UNC5D mutants

**Figure 3:**
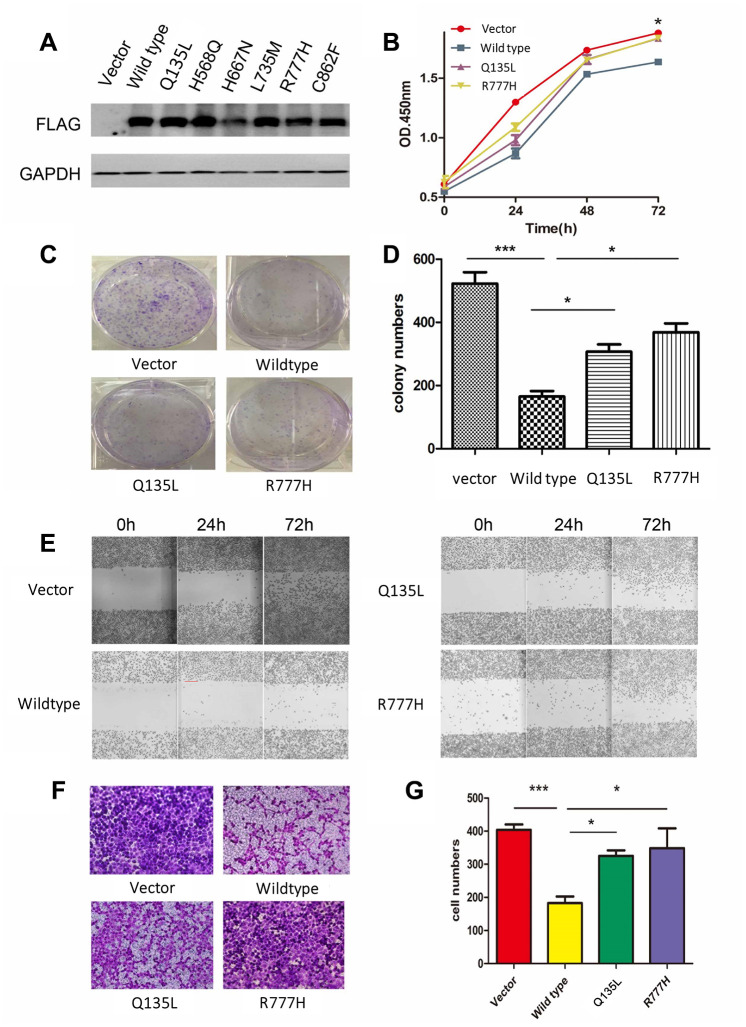
**Overexpression of *UNC5D* inhibits lung cancer growth *in vitro*.** (**A**) NCI-H1299 cells were infected with viruses harboring control vector, wild-type *UNC5D*, and *UNC5D* mutants, and protein expression was analyzed by Western blot. (**B**) The proliferation of cells overexpressing the vector, UNC5D-WT, UNC5D-Q135L, and UNC5D-R777H was determined by Cell Counting Kit-8 analysis. (**C**, **D**) Colony formation assays were conducted to evaluate the effect of UNC5D overexpression on the growth of lung cancer cells. (**E**) The mobility of UNC5D-overexpressing cells was assessed by wound healing analysis. (**F**, **G**) Cell migration analysis was determined by a Transwell assay using cells expressing the vector, wild-type UNC5D, and UNC5D mutants. *P < 0.05, **P < 0.01.

To clarify the function of *UNC5D* mutants, we analyzed the NCI-H1299 lung cancer cell line *in vitro*. Preliminary analyses demonstrated that this cell line does not express UNC5D transcripts or protein. We stably infected NCI-H1299 cells with virus harboring vectors encoding wild-type UNC5D (UNC5D-WT) or one of six UNC5D mutants (UNC5D-C862F, UNC5D-Q135L, UNC5D-H568Q, UNC5D-H667N, UNC5D-L735M, and UNC5D-R777H) or a control vector and performed Western blotting to verify that the proteins were overexpressed (Figure 3A). According to our preliminary experiment, Q135L and R777H were used to investigate the function of mutants. We next performed cell proliferation and colony formation assays and found that cell growth was inhibited by UNC5D-WT but not the mutant variants (Figure 3B–3D). Moreover, UNC5D-WT expression retarded wound closure in a scratch wound-healing assay (Figure 3E) and reduced the number of migrating cells by 2- to 3-fold in a Transwell assay (Figure 3F and 3G). These effects were not observed with overexpression of the UNC5D mutants. We further validated these effects *in vivo* using a nude mouse NCI-H1299 cell xenograft model. Consistent with the *in vitro* data, the growth of subcutis tumors expressing UNC5D-WT was inhibited (Supplementary Figure 3A), whereas tumors from cells overexpressing the UNC5D mutants had significantly larger tumor volumes (*P* < 0.05) that were similar to that of the vector control (Supplementary Figure 3B).

### *KEAP1/NFE2L2* downstream gene expression

**Figure 4:**
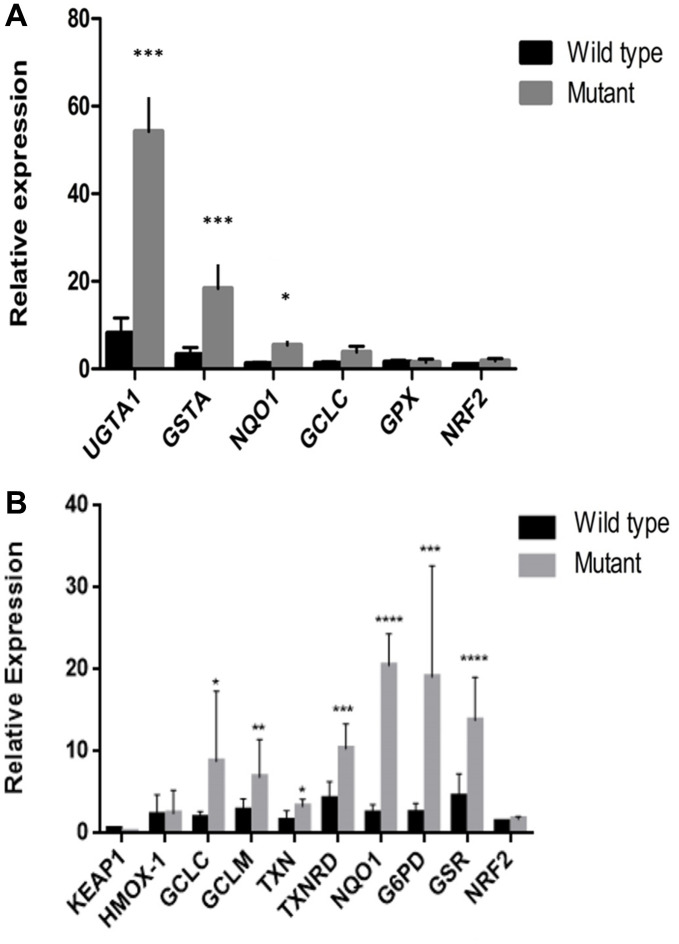
**Downstream gene expression in samples and cell lines with *KEAP1*/*NFE2L2* mutations.** (**A**) There were significant differences in *UGT1A1*, *GSTA* and *NQO1* expression between tumors with and without *KEAP1* or *NFE2L2* mutations. (**B**) There were significant differences in *GCLC*, *GCLM*, *TXN*, *TXNRD*, *NQO1*, *G6PD*, and *GSR* expression between cell lines with and without *KEAP1* or *NFE2L2* mutations. *P < 0.05, **P < 0.01, ***P < 0.001, ****P < 0.0001.

To investigate the impact of *KEAP1* or *NFE2L2* mutations in NSCLC, we compared the expression of known NFE2L2 target genes involved in oxidative stress in samples with (*n* = 9) or without (*n* = 18) either of these mutations. We found that samples with *KEAP1* or *NFE2L2* mutations had significantly increased expression of *UGT1A1*, *GSTA*, *NQO1*, *GCLC*, and *GPX* (Figure 4A). This increase was validated *in vitro*, where the expression levels of *HMOX1*, *GCLC*, *GCLM*, *TXN*, *TXNRD*, *NQO1*, *G6PD*, and *GSR* were higher in lung cancer cell lines with *KEAP1*/*NFE2L2* mutations (i.e., cell lines A549, NCI-H460, and NCI-H838) than in those without mutations (i.e., cell lines NCI-H292, 95D, SPC-A1, and NCI-H1299) (Figure 4B). These findings suggest that there may be altered responses to oxidative stress in the tumors of patients with *KEAP1*/*NFE2L2* mutations and may thus represent much-needed potential therapeutic targets for NSCLC.

## DISCUSSION

UNC5D, as a member of the UNC5 family, was identified as a netrin receptor and participated in cell migration and morphogenesis during development [27]. Previously, UNC5D was found to be a tumor suppressor and frequently downregulated in primary renal cell carcinomas [18], neuroblastoma [28, 29], bladder cancer [30] and papillary thyroid cancer [31]. Recently, Dong D et al found *UNC5D* as a putative metastatic suppressor gene that is commonly downregulated by hypermethylation in PCa [32]. Notably, we discovered *UNC5D* somatic mutations in 13 NSCLC patients and confirmed these mutations in an extended validation group of 88 patients.

Functional studies found that overexpression of UNC5D significantly decreased the cellular capacity to proliferate, migrate and invade the lung cancer cell line H1299, suggesting a tumor suppressor role of UNC5D. Additionally, further research in UNC5D mutants predicted that the mutations of UNC5D we found in NSCLC might deprive the suppression function of UNC5D. These results suggest that mutated *UNC5D* might be a driver gene in NSCLC and promote the development and progression of lung cancer. Additionally, a previous study found that UNC5D is induced during DNA damage-mediated apoptosis and is a transcriptional target of the tumor suppressor p53 [29]. However, our results revealed that *UNC5D* mutations and *TP53* mutations coexisted (Figure 1C), which suggests that the inactivation of both *UNC5D* and *TP53* could promote NSCLC. Further insight into the functional role of *UNC5D* in carcinogenesis and development may provide important information and help to identify *UNC5D* as a new therapeutic target in NSCLC.

Among other candidate drivers, we identified mutations in *PREX1* and *HECW1* that occurred at high frequencies in NSCLC. PREX1 activates insulin growth factor 1 receptor/insulin receptor signaling, as well as Rac1, phosphoinositide 3 kinase/protein kinase B, and mitogen-activated and extracellular-regulated kinase signaling, and PREX1 promotes cell and tumor viability [19]. We found 5 cases with *PREX1* gene mutations in our patients. There were two mutations located in the key functional domains DH of PREX1 and RAC1 protein interaction (Figure 1). Therefore, PREX1 mutations may be activating mutations, thereby promoting cell migration and distant metastasis in NSCLC.

HECW1, also known as NEDD4-like ubiquitin protein ligase 1, enhances the transcriptional activity of p53 and interacts with p53 to promote apoptotic cell death [33–34] and thus is considered a tumor suppressor gene in many cancers [35]. Recently, recurrent mutations in exon 11 of *HECW1* were identified in muscle-invasive transitional cell carcinoma [20]. In this study, we found *HECW1* mutations in exons 2, 7, 10 and 18 in NSCLC.

We also noted high mutation rates for the known driver genes *KEAP1* and *NFE2L2*, which encode proteins that play key roles in cell defense and survival against oxidative stress [36]. KEAP1 is tethered to cytoskeletal actin by its Kelch domain and binds with and promotes the rapid ubiquitination and degradation of NFE2L2 [37] via its functions as an adaptor protein in the Cul3-based E3 ligase complex [38]. Lignito’s results showed that loss of Keap1 or Fbxo22 induces metastasis in a Bach1-dependent manner and causes a notable increase in the metastatic phenotype in mouse models of lung cancers [39]. Furthermore, *KEAP1* and *NFE2L2* mutations were mutually exclusive in NSCLC. Singh et al. [40] first reported *KEAP1* mutations in 19% of NSCLC samples, all of which occurred within either the highly conserved Kelch domain or the intervening region domain of the protein. The rate of *KEAP1* mutations in the samples in our study was 5.9%, which is similar to the result reported by Takahashi [41]. Interestingly, most of the *KEAP1*/*NFE2L2* mutations were found in samples from patients with SCC (33.3% (10/30)). Consistent with our results, higher *KEAP1* mutation rates (60%) have been observed in ADC [42]. Our study found that UGTA1A, NQO1 and GSTA gene expression levels in lung cancer tissues carrying KEAP1/NRF2 gene mutations significantly increased in comparison with those in lung cancer tissues without KEAP1/NRF2 gene mutations. These results indicated that the KEAP1 mutations were inactivation mutations, while NRF2 mutations were activating mutations. Mutated KEAP1/NRF2 genes activate downstream oxidation reaction components (AREs), detoxification, and cell metabolism-related gene transcription levels, thereby changing cell oxidative stress levels to promote the occurrence of lung cancer.

In this study, we identified *UNC5D*, *PREX1*, *HECW1*, and *GPC5* as novel driver genes for NSCLC. We further demonstrated that recurrent mutations of *UNC5D* in NSCLC lead to a loss of tumor suppressor function *in vitro* and *in vivo*. Finally, we showed that the expression of genes involved in oxidative stress is upregulated in tumors with *KEAP1*/*NFE2L2* mutations. The above driver genes and the *KEAP1/NFE2L2* pathways represent potential therapeutic strategies for NSCLC.

## MATERIALS AND METHODS

### Patient

Tumor samples from 13 non-small-cell lung cancer (NSCLC) patients (Supplementary Table 6) were resected and H&E stained; experienced lung cancer pathologists confirmed that the samples had >80% tumor content. Adjacent normal tissues were confirmed to be free of tumor cells. *TP53* mutations were detected in all tumor samples by Sanger sequencing (Supplementary Table 5). In addition, 88 NSCLC patients were recruited from Shanghai Ruijin Hospital and the First Hospital Affiliated with Benbu Medical College as the validation cohort. Histopathologic diagnoses were established according to the World Health Organization classification and were reviewed by two independent pathologists. This study was approved by the research ethics committee of the Shanghai Ninth People’s Hospital. Written informed consent was obtained from all participants.

### Whole-exome and whole-genome sequencing

A total of 13 paired tumor and adjacent normal tissues underwent either whole-genome or whole-exome sequencing (Supplementary Table 6). Genomic DNA was extracted using a DNeasy blood and tissue kit (Qiagen, Valencia, CA, USA), and the quality of DNA was tested by using an Agilent 2100 Bioanalyzer (Agilent, Santa Clara, CA, USA). Whole-genome sequencing for seven of the paired samples was previously reported [13], and the data were obtained with an average depth of 65× coverage for tumor samples and 42× coverage for control samples. For the remaining six paired samples, sequencing libraries were prepared using a TruSeq DNA HT sample prep kit (Illumina, San Diego, CA, USA), and whole-exome enrichment was performed using SureSelect human all exon kits (Agilent) according to the manufacturer’s instructions. Paired-end sequencing (2 × 150 bp) was performed on a HiSeq 2500 sequencing platform (Illumina), as described in our previous study [13], acquiring the data with an average depth of 70× coverage for tumor samples and 67× coverage for control samples.

### Sequencing data processing and quality control

The paired sequencing data were processed as follows. First, we aligned the Illumina paired-end reads to the reference human genome hg19. Second, the mapped files (SAM format) were transformed and indexed (with SAMtools software). Next, single nucleotide variants (SNVs) and insertions/deletions (indels) were called with Genome Analysis Toolkit software (version 2.0-2). To verify the accuracy of called mutations, we filtered the mutations with the following criteria: (i) only mutations with a quality of >30 were considered; (ii) mutations with a mapping quality of <50 were excluded; (iii) a minimum of 5× coverage in the mutation variants was required; (iv) mutations with a ratio of total mapping quality zero reads to the total depth of <0.5 were considered; (v) only the variants with allelic heterozygosity of >20% were considered; (vi) the variants must be supported by both strands. To identify somatic mutations, we used adjacent normal tissues as a reference to eliminate germline polymorphisms. Somatic SNVs were annotated using ANNOVAR [43] based on the RefSeq gene database. We further filtered by (i) nonpathogenic single nucleotide polymorphisms from dbSNP 135 and SNVs from the ESP 6500 database and (ii) SNVs identified in our in-house control samples (87 healthy Chinese individuals), as described previously [13]. Overall, we identified 2,217 genes carrying 2,703 non-silent somatic mutations.

### Identify potential driver genes from NSCLC

We developed a new bioinformatics method to identify potential driver genes from a small sample size of tumor patients. First, we selected potential driver genes by comparing the mutation frequencies between NSCLC samples and healthy controls. Mutation rates of 2,217 genes in NSCLC patients were obtained from the COSMIC database. Whole-exome sequencing data of 87 individuals (made in-house) were used as the controls. We compared the mutation rates of 2,217 genes in NSCLC patients and healthy controls using Fisher’s tests, which revealed 152 genes with significant differences (*P*<0.05). Second, as novel candidate drivers, we examined six of these genes with known roles in tumorigenesis but for which no mutations have been identified. To confirm whether our methods were feasible and to identify the mutation spectrum of known driver genes in a Chinese non-small-cell lung cancer population, we also selected 21 known lung cancer driver genes that were reported in at least 2 previous studies, as well as genes with mutation rates of >2% from the COSMIC database. These 27 genes were selected for targeted sequencing in the validation cohort.

### Targeted sequencing and data analysis

Frozen tumor and adjacent normal tissues from the validation group of 88 NSCLC patients were used for targeted sequencing. Genomic DNA was extracted using the QuickGene DNA whole blood kit L (Kurabo, Japan) according to the manufacturer’s protocol. To determine the mutations of candidate genes, PCR primers were designed by iPLEX assay design software (Sequenom). Multiplexed libraries of tagged amplicons from NSCLC patients were generated by the 48×48 Access array microfluidic platform (Fluidigm) according to the manufacturer’s protocol. Deep sequencing was performed with established Illumina protocols on a HiSeq 2500 platform (Illumina). To avoid base pair variants caused by multiplex PCR, target sequences were amplified and deep sequenced in duplicate for each sample [44].

### Cell culture

HEK-293T cells and the human lung cancer cell lines NCI-H1299, A549, 95-D, NCI-H1395, NCI-H460, H292, SPC-A1 and NCI-H838 were purchased from American Type Culture Collection and cultured with RPMI 1640 medium supplemented with 10% heat-inactivated fetal bovine serum (FBS, Invitrogen). These cells were maintained at 37°C in a humidified atmosphere of 5% CO_2_.

### RNA extraction, reverse transcriptase PCR, and real-time PCR

Total RNA was extracted from lung cancer tissues and cells using TRIzol reagent (Invitrogen) according to the manufacturer’s instruction. RNA templates (1 μg) were used to synthesize cDNA with reverse transcriptase and oligo(dT) primers (Takara). Gene expression was analyzed using quantitative real-time PCR with the 2 -ΔΔCT relative quantitative method and an ABI ViiA 7 real-time PCR system (ABI). The mRNA levels of all the genes were normalized to that of the GAPDH housekeeping gene. The primer sequences used for real-time PCR are shown in Supplementary Table 7. ANOVAs and unpaired *t* tests were used for statistical analyses (the two-tailed *P* values are indicated in the figures).

### Construction of overexpression vectors and stably expressing cell lines

Full-length *UNC5D* cDNA was synthesized by Generay Biotech (Shanghai, China). To construct lung cancer cells stably expressing 3FLAG-tagged UNC5D, a lentivirus-mediated infection system was used. Briefly, 3FLAG-tagged *UNC5D* was inserted into the multicloning site of the pLenti vector. The sequences of the primers were as follows: forward, 5′-CGGGATCCCGATGGGGAGAGCGGCGGC-3′; and reverse, 5′- GCTCTAGAGCT TACTTGTCGTCATCGTCT-3′. Site-directed mutagenesis of *UNC5D* was conducted by using the Fast mutagenesis system (Transgen Biotech, Peking, China). For overexpression of the wild-type UNC5D (UNC5D-WT) and mutants, purified plasmids (pLenti-vector, pLenti-UNC5D, pLenti-UNC5D-Q135L, pLenti-UNC5D-H568Q, pLenti-UNC5D-H667N, pLenti-UNC5D-L735M and pLenti-UNC5D-R777H, and pLenti-UNC5D-C862F) were cotransfected into HEK-293T cells with packaging vectors pLP1, pLP2, and pLP/VSVG pMD2.G using Lipofectamine 2000 (Invitrogen) according to the manufacturer’s protocol. At 48 h after transfection, media containing lentivirus were collected, filtered with 0.45-μm filters, and concentrated to a viral concentration of approximately 3 × 10^8^ TU/ml. The viral particles were incubated with NCI-H1299 cells for 8 h. The infected and stably expressing clones were selected using 2 mg/ml puromycin (Sigma) and further maintained in growth medium. The overexpression of UNC5D was confirmed by real-time PCR and Western blot analysis.

### Cell proliferation

Cell Counting Kit-8 (Beyotime, China) was used to measure cell proliferation according to the manufacturer’s instructions. Each experiment was repeated at least three times. The absorbance values were measured at 450 nm on a microplate reader at 0 h, 24 h, 48 h and 72 h after treatment.

### Colony formation assay

We plated 1,000 infected NCI-H1299 cells in a 6-well plate and incubated them in medium for 10 days. Precooled methanol was used to fix colonies, and then 0.5% (w/v) crystal violet was used to stain for half an hour and counted under the microscope.

### Wound-healing assay

A 200-μl pipette tip was used to scratch the cells filled in 6-well plates, washed with phosphate-buffered saline, and incubated in RPMI 1640 medium without FBS. The distances the cells moved were determined by phase-contrast microscopy (Olympus) at the designated time points.

### Cell migration assay

Infected NCI-H1299 cells were cultured into the upper chambers of Transwell inserts with fibronectin-coated filters (8-μm pore size, Corning Life Sciences). The medium supplemented with 10% FBS was stuffed in the bottom chambers. After incubation for 20 h, cotton swabs were used to remove adhesive cells on the surface of the filter, and cells that migrated to the bottom of the membranes were fixed with methanol and then stained with crystal violet.

### Tumor xenograft model

With methods of random grouping, forty-four athymic nude mice (5 weeks old, male) (Shanghai SLAC Laboratory Animal Co. Ltd, China) were divided into four groups: vector, wild type, Q135L, and R777H. Infected NCI-H1299 cells (1 × 106) were subcutaneously injected into their right flanks, and we used calipers to measure the dimensions of the tumor every 2 days. The following formulas were used to calculate

The tumor volumes were as follows: (length (mm) × width (mm) × height (mm) × 0.5). At the end of the experiment, the animals were sacrificed, and the tumors were harvested and weighed. We performed all mouse experiments in accordance with NIH guidelines and were approved by the Shanghai Jiaotong University Animal Care and Use Committee.

### Statistical analysis

Each *in vitro* experiment was repeated at least three times. Quantitative data are presented as individual data plots or as the means ± SEM. Statistically significant differences were depended on the 2-tailed unpaired Student's t-test. Pearson or Spearman correlations were used to evaluate the correlations between gene expression and potential causative variables, and univariate analysis was performed using logistic regression. The above analyses were performed using SPSS 13.0 software (SPSS, Chicago, IL). P values < 0.05 were considered to be significant.

## Supplementary Material

Supplementary Figures

Supplementary Table 1

Supplementary Table 2

Supplementary Table 3

Supplementary Table 4

Supplementary Tables 5 and 7

Supplementary Table 6
