# Supplementary material for: Exome sequencing identifies somatic mutations in novel driver genes in non-small cell lung cancer
Source: Aging (Albany NY). 2020 Jul 6;12(13):13701–15. doi: 10.18632/aging.103500 (PMC7377869; doi:10.18632/aging.103500)
Supplement: Supplementary Figures [file aging-12-103500-s007..pdf]

SUPPLEMENTARY FIGURES

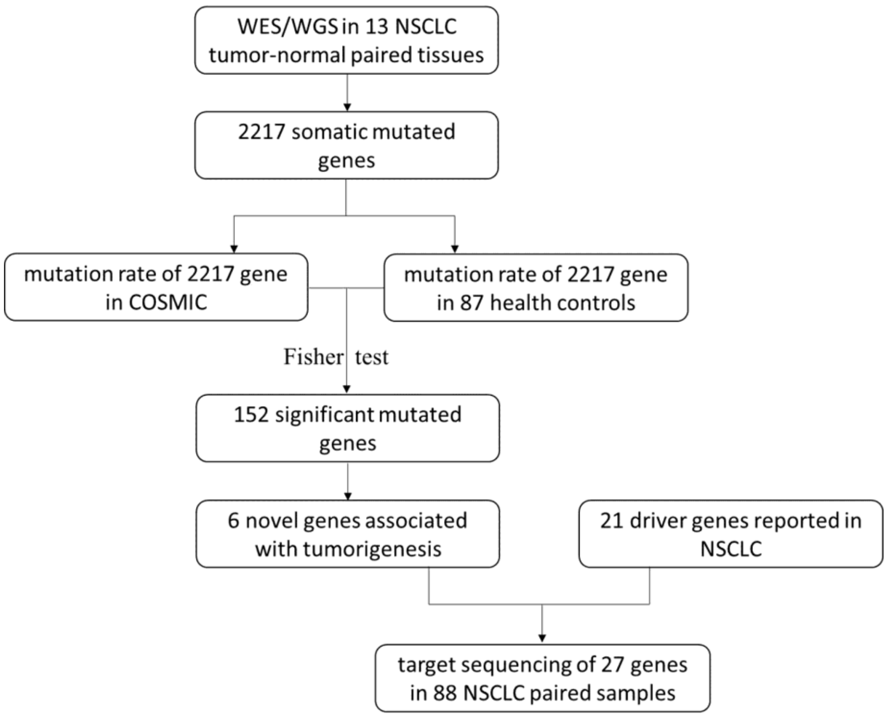

Supplementary Figure 1. Workflow for candidate gene sequencing. Thirteen paired tumor-normal tissues were collected from NSCLC patients and subjected to whole-genome/whole-exome sequencing, revealing 2,217 somatic mutated genes; the mutation rates for 152 of these genes are significantly higher in NSCLC samples (collected from the COSMIC database, version 68) than in 87 healthy controls. Fourteen genes that are correlated with tumorigenesis were selected to perform exome target sequencing among 88 tumor-normal NSCLCs.

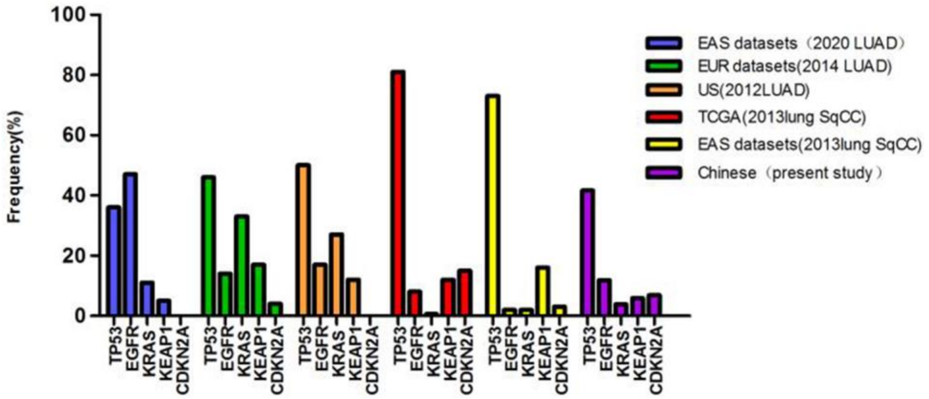

Supplementary Figure 2. Comparison of the mutation frequency of 5 known driver genes (TP53, EGFR, KRAS, KEAP1 and CDKN2A) across different large-scale genomic cohorts with lung adenocarcinoma or squamous cell lung cancers.

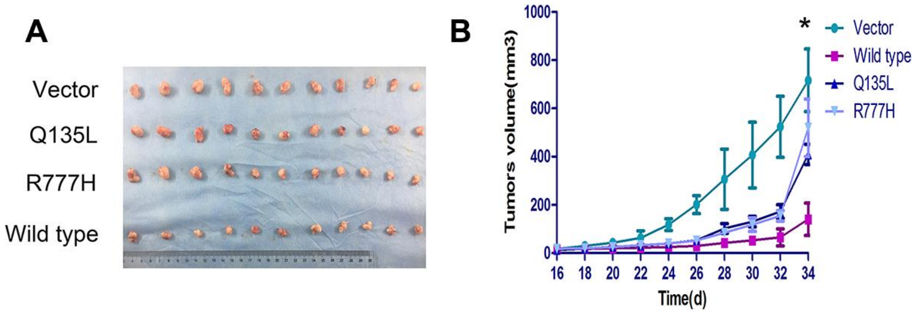

Supplementary Figure 3. Overexpression of *UNC5D* inhibits lung cancer growth *in vivo*. (**A**) A nude mouse NCI-H1299 cell xenograft model was established for the four groups. Tumors from nude mouse xenograft models after 35 days. (**B**) Growth curve of tumor volume measured on the indicated days. *$P < 0.05$.
