# Supplementary material for: Exome sequencing identifies somatic mutations in novel driver genes in non-small cell lung cancer
Source: Aging (Albany NY). 2020 Jul 6;12(13):13701–15. doi: 10.18632/aging.103500 (PMC7377869; doi:10.18632/aging.103500)
Supplement: Supplementary Tables 5 and 7 [file aging-12-103500-s003..pdf]

SUPPLEMENTARY TABLES

Supplementary Table 5. Clinical feature of 13 NSCLC patients.

Patient #	Sex	Age	Tobacco smoke		Histology	TNM	Tumor stage	WGS or WES
			Smoking status	Pack year				
823	Female	53	No	NA	AD	T2N0M0	Ⅰ B	WGS
759	Male	51	10 years	183 packs	AD	T2N0M0	Ⅰ B	WGS
781	Male	64	over 20 years	over 183 packs	AD	T1N0M0	Ⅰ A	WGS
711	Male	38	20 years	730 packs	SCC	T3N2M0	ⅢB	WGS
803	Male	60	20 years	730 packs	SCC	T1N3M0	ⅢB	WGS
829	Male	61	over 40 years	73 - 91 packs	SCC	T2N2M0	ⅢA	WGS
709	Female	62	No	NA	AD	T2N0M0	Ⅰ B	WGS
RJ-11T	Male	55	NA	NA	SCC	NA	NA	WES
RJ-19T	Male	48	NA	NA	SCC	NA	NA	WES
BB-22T	Male	49	20 years	365 packs	SCC	T3N0M0	IIB	WES
BB-16T	Female	75	No	NA	SCC	T2aN2M0	IIIA	WES
BB-43T	Male	54	30 years	730 packs	SCC	T2N1M0	IIB	WES
RJ-8T	Male	72	NA	NA	SCC	NA	NA	WES

#NA： Not available; WGS: Whole genome sequencing; WES: Whole exome sequencing.

Supplementary Table 7. Primer sequences used for real-time PCR.

Gene	F-PRIMER	R-PRIMER
GCLC	TGAAGGGACACCAGGACAGCC	GCAGTGTGAACCCAGGACAGC
GCLM	AATCTTGCCTCCTGCTGTGTGA	TGCGCTTGAATGTCAGGAATGC
GSR	ACCCCGATGTATCACGCAGTTA	TGTCAAAGTCTGCCTTCGTTGC
TXN	TTTCAGGAAGCCTTGGACGCT	GCAACATCCTGACAGTCATCCAC
TXNRD	ACGGTGATGCTGGCAATAGG	CTGGGGTGAGCTCCACCTTA
NQO1	CAGTGGTTTGGAGTCCCTGCC	TCCCCGTGGATCCCTTGCAG
G6PD	TTCATGTGGCTGTTGAGGCG	CAGTGGCTGACATCCGCAAA
NRF2	ATAGCTGAGCCCAGTATC	CATGCACGTGAGTGCTCT
HMOX1	GCTGCTGACCCATGACACCAAGG	AAGGACCCATCGGAGAAGCGGAG
KEAP1	ATTTTGGGGAGGTGGCCAAG	TTGACCCAGTTGATGCAGGC
UGT1A1	AAACGATCTGCTTGGTCACCC	CTTAGTCTCCATGCGCTTTGC
GSTA1	ATGATCCTCCTTCTGCCCGT	GGCTCAGCTTGTTGCCAAC
GPX1	CAACCAGTTTGGGCATCAGG	ATGAAGTTGGGCTCGAACCC
